# Origin of dislocation luminescence centers and their reorganization in p-type silicon crystal subjected to plastic deformation and high temperature annealing

**DOI:** 10.1186/s11671-017-2133-6

**Published:** 2017-05-19

**Authors:** Bohdan Pavlyk, Markiyan Kushlyk, Dmytro Slobodzyan

**Affiliations:** Department of Sensor and Semiconductor Electronics, Ivan Franko National University of Lviv, 107, Tarnavskoho str., Lviv, 79017 Ukraine

**Keywords:** Silicon, Dislocation-related luminescence, High temperature annealing, Thermodonors, Irradiative recombination centers in silicon

## Abstract

Changes of the defect structure of silicon p-type crystal surface layer under the influence of plastic deformation and high temperature annealing in oxygen atmosphere were investigated by deep-level capacitance-modulation spectroscopy (DLCMS) and IR spectroscopy of molecules and atom vibrational levels. Special role of dislocations in the surface layer of silicon during the formation of its energy spectrum and rebuilding the defective structure was established. It is shown that the concentration of linear defects (*N* ≥ 10^4^ cm^−2^) enriches surface layer with electrically active complexes (dislocation-oxygen, dislocation-vacancy, and dislocation-interstitial atoms of silicon) which are an effective radiative recombination centers.

## Background

Silicon has the advantage over other semiconductors in microelectronics due to availability, advanced growing, and processing technologies. The rapid development of silicon-based microelectronics requires the solution of actual problems of introduction of new optoelectronic data transmission components. However, disadvantage of silicon in comparison to direct-gap semiconductors is due to lower radiative recombination rate. Different approaches, such as impurity doping (to provide high-efficiency of internal transitions) [1], formation of silicon nanoparticles in Si [2], and use of a dislocation-related luminescence (DRL) were proposed to solve this issue.

The idea of using DRL to increase radiative recombination is very promising due to high thermal stability of luminescence centers; thus, DRL-based devices are more stable. The radiation energy coincides with a passband of fiber optics’ greatest transparency and the transparency of silicon. Furthermore, DRL centers are extremely resistant to heat treatment of samples, and consequently, based thereon devices are not practically subject of degradation [3].

Four major luminescence peaks D1, D2, D3, and D4 with the energies of 0.812 eV (1.53 μm), 0.875 eV (1.42 μm) 0.934 eV (1.33 μm), and 1 eV (1.24 μm), respectively, were determined in DRL by authors of [4] paper. Also there is a peak of band-edge luminescence (BEL) with maximum intensity at 1.1 eV (1.12 μm) [1].

It is known that high temperature annealing and elastic deformation affects on the intensity, position of peaks maxima, and shape of the DRL spectrum [5]. As the Cz–Si crystals contain high concentration of oxygen, the plastic deformation provokes so called defects clustering process (mainly oxygen precipitates) [6]. During the annealing in oxygen atmosphere *T* = 1300 K, O_I_ precipitates, which were formed after plastic deformation, acting as an additional channel of dislocations formation [7].

In papers [7, 8] it is shown that the local deformation of the subsurface layer of silicon is capable to getter defects and impurities from bulk to surface of crystal by dislocations. This type of deformation is formed as a result of lattice parameter mismatch of matrix and metal film materials. Such defects together with dislocations generate complex systems which may affect on the recombination processes in the subsurface layer of the semiconductor.

The combination of such treatment methods and technological operations determines the presence of a significant amount of linear and point defects, atoms and molecules, and their complexes in silicon subsurface layer. That is why it is important to study the mechanisms and methods of improving the DRL efficiency and the dislocation electroluminescence centers nature.

## Methods

P-type Cz–Si (silicon crystals grown by Czochralski method) crystals with resistivity *ρ* = 24 ohm • cm were used in this research. Samples were cut from a dislocation-free (*N*_d_ ≤ 10^2^ cm^−2^), single crystal silicon disk with flat surfaces orientation (111).

Formation of the contacts on the crystal surface were carried out in Vacuum Universal Post device “VUP-5” at vacuum ~ 10^−5^ Pa using nitrogen traps described in paper [9]. Aluminum film of 150 ÷ 250 nm in thickness was applied by thermal spraying (deposition rate ~15 nm/s) on the chemically polished and degassed (111) surface in a vacuum at 320 °C. Afterwards all samples were cooled down to room temperature in vacuum.

Samples prepared for electroluminescence (EL) were placed in a vacuum cryostat (with residual gas pressure of 5 • 10^−4^ Pa), and radiation recording has been performed on a modified spectrophotometer SF-20 using procedure described in paper [10]. To minimize impact of adsorbed from atmosphere atoms on the DEL spectrum, samples were subjected to vacuum annealing procedure (*T* = 430 K, *t* = 15 ÷ 20 min.). EL studies were carried out at temperature of liquid nitrogen ~ 80 K.

The experiment was conducted in three stages:

- Measurement of the electroluminescence spectra of an initial sample;
- Plastic deformation of the samples at 750 °C increase concentration of dislocations on crystals surface up to ~ 10^8^ cm^−2^ [11]. Measurements of EL and capacitance-modulation spectroscopy (CMS) spectra were conducted after the sample deformation;
- Samples annealing in flow oxygen atmosphere (FOA) at *T* = 1000 °C, 0.5 ÷ 4 h, followed by measuring the EL spectra and CMS.

To investigate the defect structure of the bulk and silicon crystal surface, the infrared absorption spectrum was carried out by using Specord M20.

Studying of deep levels was performed by CMS on samples with barrier contacts (Bi-Si-Al). For capacitance-modulation spectroscopy, besides Al contacts, Bi contacts also were formed to get barrier contact (Schottky junction). The scheme of metal contacts formation is described in paper [10].

## Results and Discussion

**Fig. 1:**
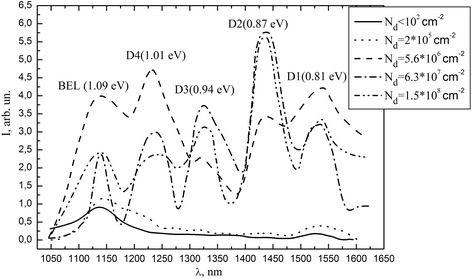
EL spectrum of samples subjected to plastic deformation with different concentration of dislocations

EL spectrum of p-Si samples with different concentration of dislocation on (111) surface after plastic deformation are represented on Fig. 1. Analysis of this research indicates that the concentration of dislocations in the range from 10^2^ to 2 • 10^5^ cm^−2^ causes no DEL in such crystals. It is connected with small quantity of radiative centers than nonradiative.

Increase of dislocation concentrations to *N*_d_ ≈ 5 • 10^6^ cm^−2^ is accompanied by increasing of radiation intensity of DEL bands (called peaks D1–D4) in the spectrum of electroluminescence [12]. Also along with this maximum of band-edge, luminescence intensity grows too.

**Fig. 2:**
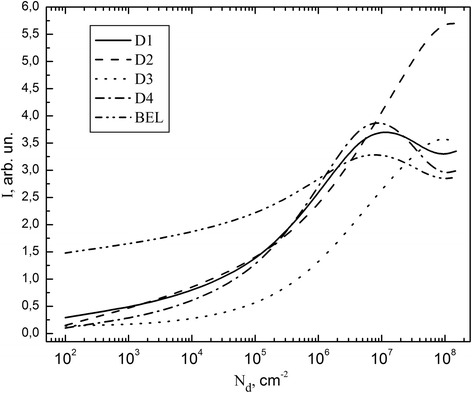
Dependence of EL peaks value (D1–D4 and BEL) of samples subjected to plastic deformation on the dislocations concentration on the surface (111) of p-type silicon

Figure 2 shows changes of electroluminescence spectrum peaks of the intensity of plastically deformed samples with different concentrations of dislocations. After analyzing of these dependencies, we can determine that the concentration of dislocations ~ 10^7^ cm^−2^ attained the saturation effect. We can also say that the changes in the concentration of dislocations have small effect on the maximum of BEL.

Increasing concentration of dislocations causes DEL intensity growth that corresponds to the literature data. Saturation is associated with balancing processes of generation and recombination (radiative and nonradiative) of free carriers in the field of dislocation core.

**Fig. 3:**
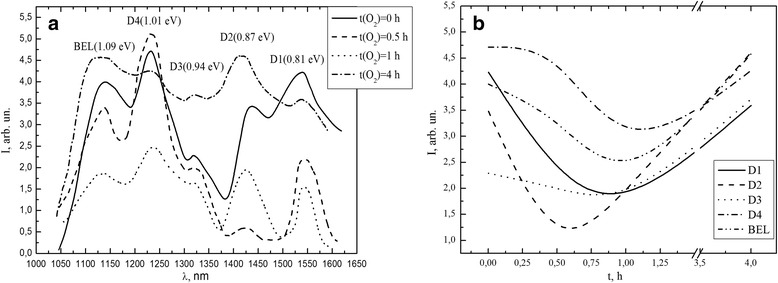
Change of EL spectrum (**a**) and change in the magnitude of peaks D1–D4 and BEL (**b**) samples subjected to plastic deformation (*N*_d_ ~ 10^7^ cm^−2^) at different time of high temperature annealing (*T* = 1000 °C) in FOA

High temperature annealing of plastically deformed silicon has a different effect on the spectrum of the electroluminescence (Fig. 3a). As it shown in paper [10], initially dominates generation of nonradiative recombination centers under the procedure of high temperature annealing.

Experimental results (Fig. 3b) demonstrate that high temperature annealing (up to 1 h) in FOA leads to a decrease of the spectrum bands (D1–D4 and BEL) intensity. These changes of EL spectrum are result of structural defect restructuring and increase of nonradiative recombination centers concentration. At the same time, we see that the increasing of annealing time causes an increase in intensity of maxima. Comparative analysis of the experimental data shows that an increase of oxygen concentration in the samples subsurface layer increases an intensity of D1 peak compared to the maximum of the band-edge luminescence (up to 15 min of annealing). This can be explained by the growth of the luminescence centers concentration related to oxygen atoms trapped by stress field around dislocations.

However, the real surface and subsurface layers of silicon exposed to much more complex adjustment related to the technological processes of samples 2 and 3 preparation. In paper [8], it is shown that the presence of linear defects significantly modified spectrum of surface states (SS) of p-Si. It is connected with actual dislocations on p-Si surface and processes of defect gettering from bulk of semiconductor to oxide film. Also important is the chemical adsorption of atoms and molecules from the atmosphere. Despite the short-term thermal desorption process during high-temperature deformation and annealing, unsaturated silicon bonds can capture atoms from the environment.

**Fig. 4:**
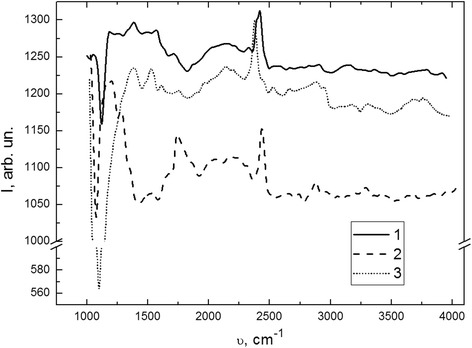
IR spectra of atoms and molecules vibrational levels in p-type silicon crystals subjected to a different type of pretreatment: *1* initial sample of p-type silicon, *2* plastically deformed sample with the concentration of dislocations *N*_d_ ≈ 10^7^ cm^−2^, *3* plastically deformed sample that was subjected to additional high temperature annealing

Studies of IR absorption spectra (Fig. 4) showed the presence on the surface of all three silicon samples groups of several peaks in the wavelength regions 1050–1200, 1250–1720, 2130–2150, 2370–2400, 2840–3300, and 3650–3800 cm^−1^. After comparison with literature data [13, 14] were established that these maximums correspond to hydrogen-, oxygen-, carbon-containing systems and their connections with silicon atoms (Table. 1).

**Table 1:** Passband maximums and corresponding defect structures obtained from a comparative analysis of vibrational spectrum of silicon samples subjected to different treatment with the literature data

Initial sample of p-Si		Plastically deformed sample		The sample annealed in oxygen after plastic deformation	
λ(max), cm^−1^	Complex	λ(max), cm^−1^	Complex	λ(max), cm^−1^	Complex
1068	O–Si–O	1042	O–Si–O	1060	O–Si–O
1203	C–C	1153	C–C	1379	C–CH_3_
1394.5	C–CH_3_	1253	Si–O–C	2136	Si–H_3_
1665	H–O–H	1352	C–CH_3_	2384	O_3_–Si–H
1720	C = O	1704	C = O	2455	C–H_2_
2126	Si–H_3_	2151	Si–H_3_	2960	
2370	O_3_–Si–H	2403	O_3_–Si–H	3193	Si–O–H
2406		2846	C–H_2_	3706	O–H
2873	C–H_2_	3170	Si–O–H		
3173	Si–O–H	3672	O–H		
3801	O–H				

Analysis of these spectra shows that plastic deformation leads to decrease in the value of transmission radiation intensity (Fig. 4, curve 2). At the same time, high temperature annealing in a flow oxygen atmosphere (an hour’s length) partially restores the original form of IR vibrational spectrum.

Experimental results show that the first plastic deformation and high temperature annealing leads to decrease in the intensity of O-Si-O maximum. After treatments, oxidation layer was etched, but due to gettering process of SiO_2_ complexes by dislocations and deformed subsurface layer of silicon crystal, their concentration grows up.

Hydrogenous complexes (C–CH_3_; Si–H_3_; O_3_–Si–H; C–H_2_) are present in all three samples, regardless of treatment type and only changing value of their peaks intensity. So as a result of plastic deformation, their concentration increases 1.5 ÷ 2.5 times, and after high temperature annealing in FOA decreases in 1.3 ÷ 1.5 times. The plastic deformation causes the hydrogenous complexes generation and their concentration in the bulk of crystals grow up. Due to high temperature annealing, these complexes diffuse into crystal volume or entrap by dislocation core. That fact causes decrease of the value of their concentrations.

In the IR spectrum of silicon crystals two bands are also represented: one about 1670 cm^−1^, the second in the range of 3400–3800 cm^−1^, which corresponds to O–H bond fluctuations in the water molecules. After high temperature annealing, these bands should disappear [13]. However, due to technological features, and the fact that water molecules are efficiently adsorbed from the atmosphere by crystal surface, the corresponding structures are always present in these spectra.

Consequently, received confirmation that an electrically neutral defect that does not participate in recombination processes that occurs in the subsurface layers of silicon crystals. However, these defects are active in the restructuring process of defect subsystem while treatment of silicon samples (plastic deformation, high temperature annealing in FOA, etc.). And also complexes O–Si–O and O–H are responsible for the absorption and scattering of dislocation electroluminescence IR radiation.

The next step was to study electrically active defect subsystem of silicon subsurface layer with dislocation and its role in the restructuring of EL centers. For this purpose, measurements of deep-level (DL) spectrum using CMS were conducted.

**Fig. 5:**
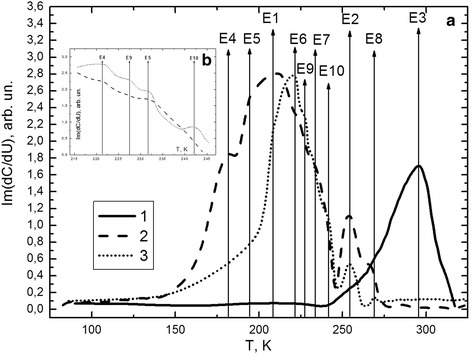
Capacitive-modulation spectrum of deep levels in the bandgap of silicon of samples at a frequency of 500 Hz (**a**): *1* initial sample of p-type silicon, *2* plastically deformed sample with the concentration of dislocations *N*_d_ ≈ 10^7^ cm^-2^, *3* plastically deformed sample that was subjected to additional high temperature annealing. **b** DLCMS graph zoomed in a range of temperatures from 215 to 245 K

Figure 5 shows the dependence of capacity imaginary part on modulation voltage from the temperature: $\mathrm{I}\mathrm{m}\left(\frac{\partial C}{\partial U}\right)= f(T)$ for diode structures Bi–Si based on samples 1, 2, and 3. For measurement of deep-level spectra with this capacitive method modulation frequency ≥ 500 Hz were used. Such frequencies enable to eliminate the influence of surface states that are not caused by defective subsystem in subsurface layer [15].

By the method of differentiation of these curves, a large number of deep levels were found. Energy occurrence of DL in energy band gap and type of corresponding defects are represented in Table. 2.

**Table 2:** Energy of deep levels and type of defect

Level label	Energy level, eV	Defect type	Level label	Energy level, eV	Defect type
E1	*E*_v_ + 0.14	Dislocation–V	E6	*E*_v_ + 0.23	Dislocation–Si_I_
E2	*E*_c_ – 0.45	Complex Fe–O	E7	*E*_v_ + 0.26	Dislocation–O
E3	*E*_v_ + 0.18	Complex C_S_–O_I_	E8	*E*_c_ – 0.15	V–O
E4	*E*_v_ + 0.04	V	E9	*E*_v_ + 0.31	C_I_–O_I_
E5	*E*_c_ – 0.08	60° dislocation	E10	*E*_v_ + 0.4	Cluster Si_I_ + Si_I_

Analysis of experimental curves showed that the original silicon subsurface layer (Fig. 5a, curve 1) contains three electrically active centers (E1, E2, and E3). Defects that are correspond to E2 and E3 energy levels based on oxygen. Also deep level E3 it is a complex of nodal carbon atom and interstitial oxygen. Oxygen and carbon are the basic background impurities in silicon crystals grown by Czochralski method. Maximum intensity of E3 peak is several times higher than E2. This demonstrates the effective gettering of oxygen by carbon atoms [16]. As for E1—value of maximum intensity is much lower, and this defect corresponds to a dislocation-vacancy complex. Since the vacancies in silicon crystals are quite mobile at temperatures above 80 K [17], it can be defined only in combination with other impurities or defects by using capacitive methods. These defects could be dislocations that occur in the surface layer of silicon at the interface of Si–SiO_2_ [18].

Plastic deformation of monocrystalline silicon leads to a significant restructuring of electrically active centers in the subsurface layer of the semiconductor. Increase of signal intensity of E1 and E2 peaks (Fig. 5a, curve 2) is connected with oxidation of crystals during deformation. Moreover, the result of this process is the generation of a significant number of dislocations (*N* = 10^7^ cm^−2^). These types of defects are the basis of new defective systems E5, E6, and E7. As for the level E4, there are difficulties in its interpretation, because its ionization energy is negligible (*E*_v_ + 0.04 eV). As Lukjanitsa`shows in paper [17], this energy level maybe responds to the vacancy defects. Since the process of plastic deformation increases the intensity of diffusion of impurities and defects from the surface into the volume of silicon, it is also possible to reverse processes: diffusion of defects by dislocations to the surface and localization in the surface layer [7]. As the concentration of linear defects on the crystal surface increased by five orders of magnitude, then occurrence probability of dislocation and point defect complexes may also increase. This is confirmed by appearances of E6 and E8 DL’s corresponding to the dislocation—Si_I_ and oxygen—vacancy centers. As for E3, it disappears on the spectrum of deep levels because there is redistribution between defective subsystems of the crystal lattice, which leads to the appearance of new centers. However, the area under the curve 2 (Fig. 5a) increases by three times that indicates the generation of electrically active defects not only due to the restructuring of existing, but also largely due to the technological treatment of silicon crystals.

Additional annealing in an oxygen atmosphere caused the appearance of new “deeper” defects and reducing of the concentration of existing complexes (Fig. 5a, curve 3). In particular, signal intensities of E1, E2, E4, E5, and E8 DL reduced. This may be caused by the formation of more complex systems. So, E4 level corresponds to the vacancies and E5 is the dislocation. High temperature annealing in an oxygen atmosphere should encourage the growth of vacancy diffusion and their interaction with dislocations or oxygen and generation of appropriate centers. However, the intensities of E1 and E8 maximums that correspond to such defects are also reduced. That indicates the presence of another mechanism of defect subsystem redistribution in the subsurface layer of silicon crystals.

However, the intensities of E6 (dislocation-Si_I_) and E7 (dislocation-O) deep levels increase (Fig. 5b). Change of E7 peak signal intensity can be caused by capturing oxygen atoms by dislocations from the environment. As for the level E6, the additional high temperature annealing increases the probability of silicon interstitial atoms diffusion and captures them on dislocations.

Furthermore, this effect make possible of complexes with their own interstitial atom formation in silicon crystal subsurface layer. One of these centers is Si_I_–Si_I_, which corresponds to DL E10 (Fig. 5b).

However, the temperature annealing can lead to the generation of electron-hole pairs in SiO_2_. Thermalized holes can get to the energy level near *E*_v_ [8], and free electrons leave insulator. They may be captured by the interstitial silicon atoms and change charge state of Si_I_. It will cause them to transition from tetrahedral positions to the hexagonal and vice versa [17]. Migration of interstitial atoms of silicon can cause displace of carbon atoms in defective complex C_S_–O_I_ and formation C_I_–O_I_ [15]. The result of this mechanism is the appearance of E9 energy level on spectrum of DL.

We perform a comparison of electroluminescence spectra (Fig. 1) and CMS of deep levels (Fig. 5) of structures based on p-type silicon before and after high temperature annealing in oxygen atmosphere. You may see the rearrangement of the intensity amplitude in favor of D1 peak. The value of this maximum increases compared to the intensity of band-edge luminescence. At the same time, we can see decrease in the amplitude of the overall peaks. Such correlation allows us to associate band D1 with the transitions from E5 = (*E*_c_ − 0.08 eV) to E7 = (*E*_v_ + 0.26 eV) energy levels. Increase of D1 maximum intensity explains by increasing in the concentration of dislocations and dislocation oxygen complexes.

E10 energy level that appears after annealing of samples, according to [17, 19, 20], corresponds to clusters of interstitial silicon atoms (Si_I_ + Si_I_). These clusters are responsible for nonradiative recombination in silicon crystals. The growth of their concentration in annealed samples may explain the overall decrease in the intensity of the electroluminescence. As for D3 and D4 maxima of dislocation, luminescence can be linked to transitions *E*_c_ → E1 (*E*_v_ + 0.14 eV) and *E*_c_ → E6 (*E*_v_ + 0.23 eV), respectively. These levels are associated with the interaction of dislocations with: vacancies (E1) and interstitial silicon atoms (E6).

## Conclusions

All of these point to a complex interaction of impurities and dislocation emitting centers under the influence of thermal annealing and elastic deformation. Such interaction behaves like incorporation of impurity atoms in the structure of these centers as well as the formation of complexes “dislocation-impurity” in which the mutual configuration of the defect determines the energy of optical transitions. Such defects could have a rather complicated structure, which varies during the heat treatment. Also it should be noted that the point defects (impurity atoms and intrinsic defects), which effectively generated at various influences on the sample participate in the processes of transformation of the extended defects and dislocation-related luminescence centers.

An analysis of IR spectra shows a high capacity of Si surface to absorb atoms from the surrounding atmosphere during plastic deformation or annealing and forming a number of complexes with oxygen, carbon, and hydrogen atoms.

High temperature annealing of silicon in an oxygen atmosphere accompanied by the formation and increasing concentrations of oxygen-dislocation complexes and thermodonors, diffusion of interstitial silicon atoms to the crystals surface, and formation of C_I_–O_I_ complexes by Watkins mechanism.

Analysis of structural defects restructuring processes using DLCMS techniques in comparison with EL spectral dependences allowed us to show that the energy levels *E*_v_ + 0.08 eV (60° dislocation), *E*_v_ + 0.14 eV (dislocation-V), *E*_v_ + 0.23 eV (dislocation-Si_I_), *E*_v_ + 0.26 eV (dislocation-O) correspond to dislocation luminescence centers.

## Abbreviations

BEL: Band-edge luminescence
CMS: Capacitance-modulation spectroscopy
*Cz*-Si: Silicon crystals grown by Czochralski method
DEL: Dislocation-related electroluminescence
DL: Deep level
DLCMS: Deep-level capacitance-modulation spectroscopy
DRL: Dislocation-related luminescence
EL: Electroluminescence
FOA: Flow oxygen atmosphere
IR: Infrared
SS: Surface states
